# A New Style of Dimethylnitrosamine Induced Fulminant Hepatitis in Mice

**DOI:** 10.5812/hepatmon.12901

**Published:** 2013-09-14

**Authors:** Hanping Liu, Qingya Li, Hong Liu, Yuansheng Wu, Jinyang He

**Affiliations:** 1 Tropical Medicine Institute, Guangzhou University of Chinese Medicine, Guangzhou, China; 2 The first Affiliated Hospital of HeNan University of Traditional Chinese Medicine, Zhengzhou, China; 3 The second Affiliated Hospital, Guangzhou University of Chinese Medicine, Guangzhou, China; 4 Photonic Chinese Medicine, College of Biophotonics, South China Normal University, Guangzhou China

**Keywords:** Liver, Mice, Dimethylnitrosamine, Hepatitis

## Abstract

**Background:**

There is still no suitable mice model that can completely mimic the human fulminant hepatitis, which sets a block for drug effect evaluation and mechanism researching of human fulminant hepatitis.

**Objectives:**

The aim of this study was to establish an animal model able to mimic the main features of human fulminant hepatitis.

**Materials and Methods:**

Dimethylnitrosamine (DMN) was peritoneally injected to mice for liver injury induction. Serum biochemicals, and Prothrombin Time were tested, and Prothrombin activity was calculated, the liver tissue pathological changes were evaluated via macroscopic view observation, HE staining, immunochemical staining, and electron microscopy observation. The mRNA levels of TNF-a, Fas, and IL-1beta were tested with quantitative PCR assay.

**Results:**

The serum levels of both ALT and AST were elevated significantly and showed a high plateau. Liver pathological changes were progressed before 48 hours post DMN injection and then started to restore. The mRNA and protein expression levels of TNF-α and IL-1β were significantly elevated. The PT started to extend from 36 hours and PTA was lower than 40% from then on.

**Conclusions:**

This kind of DMN induced mice liver injury is similar to human fulminant hepatitis in main features. This work provided a mice model which could mimic human fulminant hepatitis, and could be valuable for fulminant hepatitis mechanism research and liver protection drug evaluation.

## 1. Background

Fulminant hepatitis (FH) is characterized by the development of severe liver injury with impaired synthetic capacity and encephalopathy in patients with previous normal liver function or at least well compensated liver disease (1). The marked feature is prothrombin time (PT) less than 40% of the standardized values (2). The FH occurs as a result of functional failure of a large part of the hepatic parenchyma, and severity is proportional to the level of hepatic damage.

There were few reports of liver histological features of human FH because invasive procedure with a risk of internal bleeding is usually not indicated. Most of the reports about Liver pathological changes of human FH were from patients dying of FH (3-6). The reported FH liver pathological changes include acute hepatocyte necrosis and liver inflammation of varying degrees, cholestasis, lymphocytic cholangitis, and Kupffer cell prominence and so on (3, 6). Therefore, the detailed literature of histological features of FH is still a great need. Because of the risk of internal bleeding and high death rate, liver biopsy should be conducted on FH animal model if we need to learn more about the liver pathological changes of human FH. Thus, there is an urgent need for an animal model which could resemble human FH in main features.

FH is mainly caused by viruses in Asia (2, 7) and drug in America in the past time (8, 9)，while drug has now overtaken viruses (particularly hepatitis B virus) as the leading cause of FH due to developments in antivirus drug therapy (7). Therefore, mechanism of drug induced fulminant hepatitis is in great need for elucidation. However, there is no suitable animal model that can typically mimic the human FH up to now.

DMN was used for liver injury or fibrosis modeling from decades ago (10-12). Different doses style of DMN injection can cause different patterns of liver pathological changes. Multidose DMN injections cause rat liver fibrosis, while single dose causes liver injury (11, 13). DMN is a potent hepatotoxin, carcinogen, and mutagen. DMN-induced liver injury in rats seems to be a good animal model for early liver cirrhosis (14). A model of cirrhosis induced by chronic, discontinuous treatment with a low dose of DMN in rat has been reported to reproduce a number of characteristics of this liver disease (11). The extent of liver injury can be easily estimated by measuring the activities of certain plasma enzymes, e.g. alanine aminotransferase (ALT), and aspartate aminotransferase (AST).

Previous study had described that a single does (30mg/kg) i.p. administration of DMN induced severe and fatal toxicity in liver tissues in mice which resembled human fulminant hepatitis (10). But this kind of liver injury model cannot be used for drug effect evaluation because all of the mice would die within 48 hours.

## 2. Objectives

In the present study, we show a new style of DMN induced mice liver injury model in which most of mice can survive through a single dose of DMN injection for more than a week, and show pathological changes similar to the human being‘s FH. The PT was extended significantly post DMN injection which is also similar to human FH. Moreover, the mRNA and protein of both Interleukin- 1β (IL-β) and TNF-α were elevated, and TNF-α may play a critical role in the DMN induced mice FH.

## 3. Materials and Methods

### 3.1. Animals

Kunming Mice, 20 ± 2g, male, were purchased form experimental animal center of Guangzhou University of Chinese Medicine. The mice were housed in SPF grade animal room, and settled in plastic cages in the experimental animal center of Guangzhou University of Chinese Medicine. They were fed with standard mouse diet and water according to the guidelines approved by the China Association of Laboratory Animal Care.

### 3.2. Model Making and Samples Collection

50 mice were peritoneally injected with a single dose of 15 mg/kg DMN (Tianjin Chemical Reagent Research Institute, China) solution in 0.9% NaCl. Serum and liver tissue samples were collected at the time points of 0, 6, 12, 24, 36, 48, 72, 96 and 120 hours post DMN injection. Four mice were killed at every time point for samples collection.

### 3.3. Serum Biochemical Items Testing

The levels of serum alanine aminotransferase (ALT) and aspartate amino-transferase (AST) were measured using testing kits of an ultraviolet method. Albumin levels were measured with a testing kit of bromocresol green method; total protein levels were measured with a testing kit Biuret method. All of the testing kits were from the NanJing Jiancheng Bioengineering Institute.

### 3.4. Liver Index Calculation

The mice whole body and liver were weighted respectively when killing them. The Liver Index was calculated according to the formula: Liver Index = liver weight (mg)/whole body weight (g).

### 3.5. Histopathology

The whole liver was excised for observation and picture was taken. Part of the liver tissue was fixed with 4% paraformaldehyde. The fixed liver tissues were then paraffin embedded and cut. Paraffin sections were stained with hematoxylin and eosin (HE) for routine examination and stained for the expression of TNF-α (TNFα Antibody (52B83), 1:200;Santa Cruz Biotechnology, Santa Cruz, The USA) and IL-1β (IL-1β Antibody (H-153) ,1:200; Santa Cruz Biotechnology, Santa Cruz, The USA) for protein expression detection. Immunoglobulin G was used as a negative control instead of primary antibodies. The immunohistochemical staining was conducted using the streptavidin-biotinylated peroxidase complex (S-ABC) method. A part of liver tissue was fixed with glutaraldehyde in 0.2 mol/L phosphate buffer, and embedding in epon/araldite for electron microscopy.

### 3.6. Quantitative Determination of the Liver Injury

Semiquantitative assessment of liver injury in each group was evaluated by the area of liver necrosis on the whole slide in each group (15). 0 for no necrosis; 1 for < 10% area of necrosis; 2 for 10%-30% area of necrosis; 3 for 30%-50% area of necrosis.4 for > 50% area of necrosis； all the evaluations of liver damage were conducted by two independent observers. The average score of three mice in each group was taken as the score for that group.

Each Immunohistochemical stained section was examined by two independent observers according to the severity of the staining. The severity of the staining was assigned a score of 0 (no staining), 1 (< 10% of cells or extracellular environment staining within a disease focus), 2 (10% - 30% of cells or extracellular environment staining within a disease focus), 3 (30 - 50% of cells staining or extracellular environment staining within a disease focus ), or 4 ( > 50% of cell staining or extracellular environment staining within a disease focus) (16, 17).

### 3.7. Liver Tissue mRNA Testing

The total RNA was extracted from the liver tissues using TRIzol reagent (Life Technologies, Grand Island, NY). The first-strand cDNA was synthesized using the M-MLV First-strand Synthesis Kit (Invitrogen, The USA). SYBR Green Quantitative PCR assay was used for detection of TNFα、Fas、IL-1β, and glyceraldehyde-3-phosphate dehydrogenase (GAPDH).

The quantitative PCR was performed using SYBR Green qPCR SuperMix-UDG reagent (Invitrogen, The USA), and an ABI 7300 quantitative PCR system (Applied Biosystems, The USA). The relative quantity was normalized to the GAPDH internal.

### 3.8. Prothrombin Time Testing

Blood samples were collected with sodium citrate as an anticoagulant. Plasma was isolated for testing. Prothrombin Time testing Kit from DEAE Company was used. SYSMEX CA6000 Thrombelastograph Hemostasis Analyzer from Japan was used for PT analysis. The method for PTA calculation was as follows: PTA = [control PT - (control PT × 0.6)］÷［Model PT - (control PT × 0.6)］× 100%. The Control PT was the average of blank control group; Model PT was the PT of other group.

### 3.9. Statistical Analysis

The statistical analysis was performed with SPSS 11.0 software. Student’s t-test with 95% confidence bounds was used for the two groups comparison; Significant differences were defined as a P < 0.05.

## 4. Results

### 4.1. Mental Status and Death Rate

The mice started to dispirit from 12 hours post DMN injection and continued to the end of observation. The first dead mouse was found between 24-48 hours. The second occurred between 48-72 hours, and the third occurred between 96-120 hours. Another 2 mice were died between 120-168 hours. Therefore, this style of DMN induced mice liver injury can survive for up to 1 week and only 10% of mice would die.

### 4.2. Serum ALT/AST and Total Protein and Albumin Results

Both the ALT and AST levels are elevated at all of the time points after DMN injection. Their dynamic changes are presented in Table 1. 

The total protein level seemed to decrease from the onset to the 36 hours and then slowly increased at the remained time points after DMN injection. The Albumin levels were slightly increased at 6 hours, and then slowly decreased at 12 hours to 72 hours slightly and increased at 120h post DMN injection (Table 1). 

**Table 1.** Serum ALT, AST, Total Protein, Albumin Levels, and Liver Index of DMN Induced Mouse Liver Injury.

	Number	0h	6h	12h	24h	36h	48h	72h	120h
**ALT U/ml**	4	2.873 ± 1.061	7.097 ± 3.142 ^b^	55.019 ± 36.001 ^b^	59.846 ± 24.058 ^b^	60.594 ± 7.275 ^b^	76.869 ± 11.893 ^b^	15.912 ± 10.3 ^b^	24.804 ± 13.176 ^b^
**AST U/ml**	4	32.3 ± 6.087	44.1 ± 2.061^a^	157.275 ± 60.945 ^b^	146.65 ± 52.634 ^b^	145.9 ± 16.18 ^b^	153.125 ± 33.553 ^b^	58.1 ± 21.876 ^b^	69.3 ± 27.495 ^b^
**Total protein g/L**	4	431.205 ± 22.095	343.3 ± 94.652	308.903 ± 13.864 ^b^	296.088 ± 61.727 ^b^	236.811 ± 45.805 ^b^	281.7 ± 32.311 ^b^	287.095 ± 25.195 ^b^	331.834 ± 69.623 ^a^
**Albumin g/L**	4	96.166 ± 2.703	104.089 ± 6.12	99.429 ± 14.015	88.709 ± 19.693	78.3 ± 6.317 ^b^	70.532 ± 5.285 ^b^	70.066 ± 5.258 ^b^	81.407 ± 8.977 ^b^
**Liver Index**	4	5.12 ± 0.35	5.26 ± 0.29	5.17 ± 0.27	5.42 ± 0.41	3.82 ± 0.20 ^b^	2.91 ± 0.27 ^b^	3.77 ± 0.26 ^b^	3.54 ± 0.22 ^b^

^a^ P < 0.05

^b^ P < 0.01.

### 4.3. Liver Index Results

The Liver Index remained no marked difference within 0-24 hours post DMN injection. Then the Liver Index decreased at all the following time points. The lowest Liver Index occurred at 48 hours post DMN injection (Table 1). 

Table 1 showed that both the ALT and AST were elevated significantly at all the observed time points post DMN when compared to 0 hour (before DMN injection). They seemed presented as a high plateau between time points of 12 and 48 hours. The serum total protein levels were significantly decreased from 12 to 120 hours compared to 0 hour. The Albumin levels were significantly decreased from 36 to 120 hours compared to 0 hour. The liver index was significantly decreased from 36 to 120 hours compared to 0 hour. 

### 4.4. HE Stain Results

**Figure 1.**
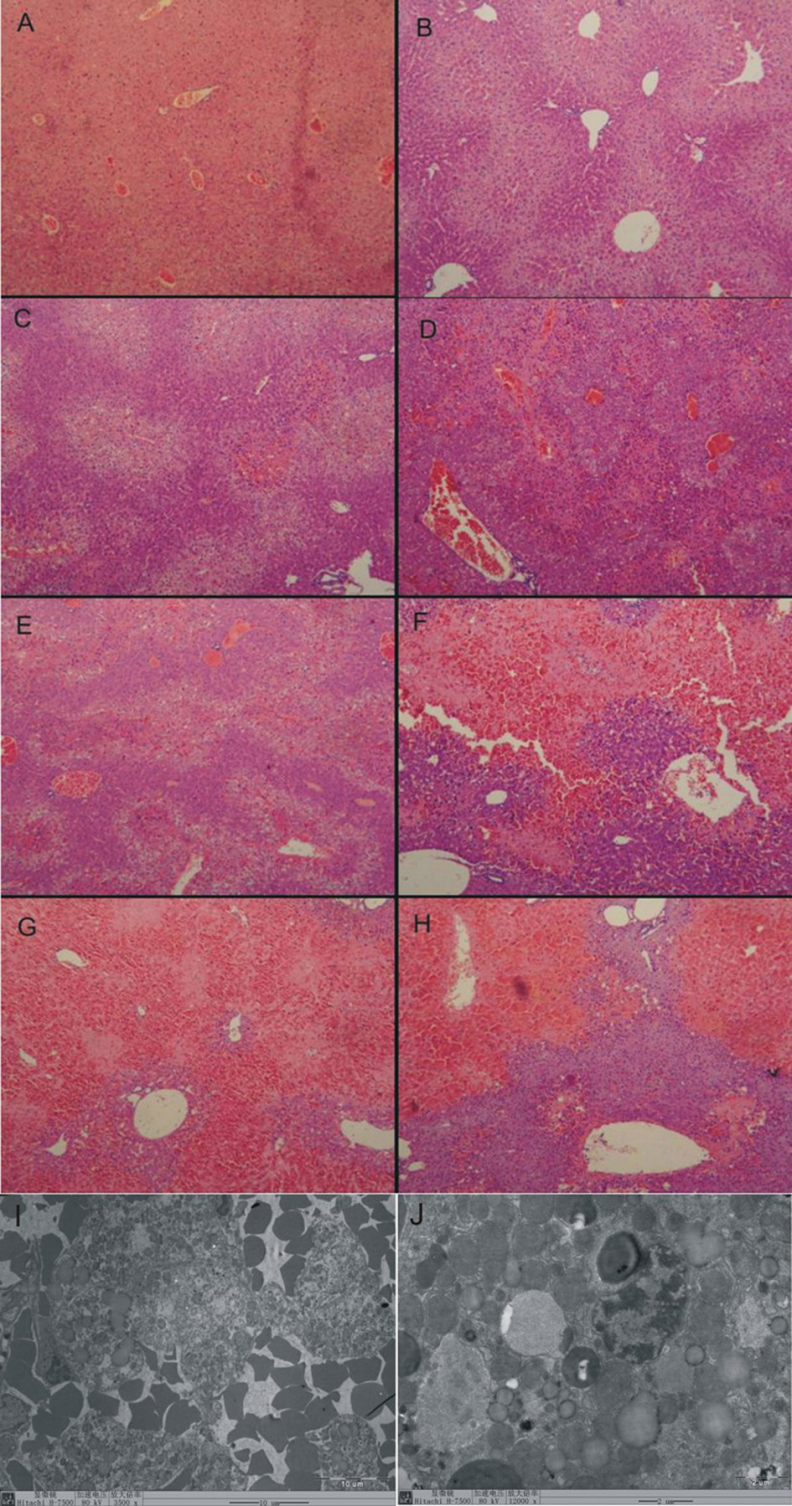
HE Staining and Electron Microscope of Liver Tissue in DMN Induced Mouse Liver Injury (HE staining：100 ×；Electron microscope：8000 ×)

At 0 hour, the normal liver tissue (Figure 1 A) showed regular arranged lobular structure， no bleeding, no liver cell degeneration and necrosis, and no fat deposition. 

At 6 hours， the vacuolar degeneration in hepatocytes was appeared, which mainly located in liver parenchyma among the blood vessels (Figure 1 B). 

At 12 hours vacuolar degeneration in hepatocytes was severer, and marked bleeding was appeared. There was an extensive hemorrhage and necrosis disease focus surrounded by inflammatory cell occurred in some area (Figure 1 C). 

At 24 hours the extensive hemorrhage and necrosis disease focus spread over the liver tissue. The lobular structure disappeared within the necrosis disease focus which surrounded and invaded by big quantity of inflammatory cells (Figure 1 D). 

At 36 hours the extensive hemorrhage and necrosis disease focus distributed densely over the liver tissue. Vacuolar degeneration appeared at the edge of the necrosis disease focus. There were big lipid drops deposited in the hepatocytes (Figure 1 E). 

At 48 hours the extensive hemorrhage and necrosis disease focus expanded to the whole sight. The lobular structure disappeared in most of the hemorrhage and necrosis disease focuses (Figure 1 F). 

At 72 hours most of the liver tissues were occupied by the hemorrhage and necrosis disease focuses. (Figure 1 G). 

At 120 hours the extensive hemorrhage and necrosis disease focuses also occurred in most of the liver tissues. Inflammatory cells infiltration decreased than before (Figure 1 H). The liver injury score is listed in Table 2.

**Table 2.** Liver Tissue mRNA Levels of TNF-a，Fas and IL-1βin DMN Induced Mouse Liver Injury

	Number	0h	6h	12h	24h	36h	48h	72h	120h
**TNF-α**	4	1.79 ± 0.59	15.4 ± 3.49 ^b^	16.9 ± 3.19 ^b^	8.42 ± 2.68 ^b^	3.44 ± 0.96 ^a^	5.29 ± 1.95 ^b^	4.02 ± 0.92 ^b^	4.82 ± 1.11 ^b^
**Fas**	4	8.83 ± 2.31	5.51 ± 1.57	6.13 ± 1.42	8.73 ± 1.98	8.98 ± 1.93	8.85 ± 1.88	8.26 ± 2.49	8.1 ± 2.12
**IL-1β**	4	3.24 ± 1.09	12.45 ± 1.65 ^b^	13.09 ± 1.66 ^b^	18.84 ± 2.3^b^	23.34 ± 3.98 ^b^	29.11 ± 2.83 ^b^	18.22 ± 2.70 ^b^	21.32 ± 4.05 ^b^

^a^ P < 0.05

^b^ P < 0.01

### 4.5. Electron Microscope Results

The electron microscope view showed 48 hours post modeling. Great amounts of red cells invaded into the liver tissues (Figure 1 I). Lipid drops deposited in parts of hepatocytes (Figure 1 J). The mitochondrial swelling occurred in lots of hepatocytes (Figure 1 B). Small amounts of hepatocytes were progressing into apoptosis. 

### 4.6. Immunohistochemical Staining

#### 4.6.1. TNF-alpha Immunohistochemical Staining

At 0 and 6 hours， the mice liver tissues showed slightly stained brown color in cytoplasm of few hepatocytes which was mainly around the blood vessels. At 12 and 24 hours， the brown color was deeper and mainly located in the disease focuses. At 36 and 48 hours， the range of the brown color was expanded and the deepest brown color appeared. At 72h，the deep brown color expanded and accompanied with disease focuses. At 120 hours， the deep brown color was still located in the disease focuses but seemed to be reduced than before. The TNF-α Immuno-staining grade is listed in table 2.

#### 4.6.2. The IL-1β Immunohistochemical Staining

The blank control mice liver tissues did not show marked brown color. At 6 hours， the slightly positive brown color appeared around blood vessels. The brown signal increased at 12 hours and then expanded and changed deeper at 24 hours. The brown signal continued to expand and got deeper at 36 and 48 hours. At 72 and 120 hours， the brown signal appeared a bit lighter than before but still outstanding. The IL-1β Immuno staining grade was listed in Table 3. 

**Table 3.** The liver Injury HE Staining Score and TNF-α and IL-1β Immunohistochemical Staining Score of the MDN Induced Mouse Liver Injury

Time points	0h	6h	12h	24h	36h	48h	72h	120h
**Liver injury score (HE stain)**	1	1	2	2	3	4	4	4
**TNF-α Immunohistochemical staining score**	2	2	3	3	4	4	4	4
**IL-1β Immunohistochemical staining score**	0	2	2	3	3	4	4	4

Table 3 showed that the HE stain for the liver injury score had no marked liver injury and scored as 1 at time points of 0 and 6 hours. The time points of 12 and 24 hours showed slight injury and were scored as 2. The time point of 36 hours, showed severer injury than before and was scored as 3. The time points of 48, 72, and 120 hours showed severest injury and were scored as 4. The TNF-α Immunohistochemical staining signal had positive results at time points of 0 and 6 hours and were scored as 2. The signals were enhanced at time points of 12 and 24 hours, and were scored as 3. The signals were strongest at time points of 48, 72, and 120 hours, and were scored as 4. The IL-1β Immunohistochemical staining showed no positive findings at time point of 0 hour and was scored 0. The positive signals were occurred at time points of 6 and 12 hours, and both the two time points were scored as 2. The signals of time points of 24 and 36 hours were stronger than before, and were scored as 3. The remained time points of 48, 72, and 120 hours showed strongest signals and were scored as 4. 

### 4.7. Macroscopic View

The normal liver tissue showed smooth surface with red and brown color. At 6 and 12 hours， the liver appeared normal but small granules could be seen by naked eye. The liver showed slight blood stasis at 24 and severer at 36 hours， the color changed to deep red. 3 of 4 mice showed edema. At 48h, the liver started to shrink and all of the 4 mice showed edema. The liver tissue still showed deep red color. The liver still showed blood stasis and shrink markedly at 72 and 120 hours. All of the mice in both time points showed edema.

### 4.8. mRNA Results

The TNF-α mRNA was significantly elevated at 6 hours. The highest level was appeared at 12 hours and then slightly decreased but remained high level until 120 hours. The variation tendency of IL-1βmRNA levels was similar to the TNF-α. The Fas mRNA levels did not show significant changes during the observation period (Table 3). 

In Table 3 the quantitative PCR testing showed that the TNF-αmRNA levels of all the time points post DMN injection were significantly elevated when compared to 0 hour. The Fas mRNA levels of all the time points post DMN injection had no significant changes when compared to 0 hour. Similar to the TNF-α, the IL-1β mRNA levels of all the time points post DMN injection were significantly elevated when compared to 0 hour. 

### 4.9. Prothrombin Time Testing

The Prothrombin Time (PT) of normal mice averagely was 9.36 seconds. It was started to significantly extend from 36 hours post DMN injection. Then at 48 hours， the Prothrombin Time reached 21.25 seconds, which is the longest in the observed time points. Then at 72 and 120 hours， the PT was 17.1 and 14.7 seconds respectively. The prothrombin activity (PTA) was calculated from prothrombin time. The PTA was started to decrease from 24 hours and reached the lowest value at 48 hours. The PTA of 36, 48, and 72 hours were lower than 40% (Table 4). 

**Table 4.** PT and PTA Levels of DMN Induced Mouse Liver Injury

	Number	0h	6h	12h	24h	36h	48h	72h	120h
**PT(seconds)**	9.36 ± 1.17	8.25 ± 0.85	8.53 ± 0.80	10.55 ± 1.36	16.05 ± 3.46 ^a^	21.25 ± 4.06 ^b^	17.1 ± 2.51 ^b^	23.15 ± 3.18 ^b^	14.7 ± 2.34 ^b^
**PTA**	100%	142%	128%	75.88%	35.88%	23.95%	32.60%	17.53%	41.17%

^a^ P <0.05

^b^ P < 0.01.

Table 4 showed that the PT was extended significantly between the time points of 24 and 120 hours when compared to 0 hour. The PTA was started to decrease from 12 hours and was lower than 40% between the time points of 24 and 72 hours. 

## 5. Discussion

There are several kinds of fulminant hepatitis mouse models currently in use. concanavalin A (ConA)-induced hepatitis mouse model is mediated by T cells apoptosis (18, 19), in which the liver injury culminated at 8 hours post ConA injection, and then restore within 24 hours. Therefore, ConA-induced mice liver injury model may not be suitable for the liver protection affect evaluation drugs which need several time of administration. Additionally the Con-A induced liver injury mice model does not show the main features of humans fulminant hepatitis.

The LPS can induce fulminant liver hepatitis in D-GalN-sensitized mice (20). The hepatocytes death of this kind of mice fulminant hepatitis model is mainly mediated by TNF-a which subsequently leads to hepatic necrosis (21, 22). But most of the mice would die within 12 hours post modeling (23). We showed here that the DMN induced mice liver injury resembles in main features of human fulminant hepatitis. For the macro view, the changes of liver surface, volume and occurrence of edema are almost the same as human fulminant hepatitis.

Both the ALT, AST are elevated post modeling. The total protein and albumin are both decreased. The most critical pathological changes of liver tissues are necrosis accompanied with liver inflammation which is similar to the human FH. The liver tissue pathological changes deteriorate with the time going, and culminated at 48 hours post DMN injection. This would provide a space for several times of drug administration. Therefore, DMN induced mice fulminant hepatitis can be used for drug effect evaluation especially for oral drugs.

The mRNA testing in liver tissue showed that TNF-α but not Fas is dramatically elevated in DMN induced mice FH; The TNF-α immunohistochemical staining results also showed that the TNF-alpha protein was dramatically elevated. The mRNA of Fas was not elevated which suggests that the TNF-α may play a critical role in the DMN induced mice FH.

The PT was extended significantly after DMN injection and PTA was lower than 40% at the time points of 36, 48, and 72 hours post DMN injection. This also resembles that of human FH.

Collectively this DMN induced mice FH are similar to human FH. This mouse model can be used for evaluating oral drugs for fulminant hepatitis treatment or for mechanism research of fulminant hepatitis.
